# Long-Term Human Hematopoietic Stem Cell Culture in Microdroplets

**DOI:** 10.3390/mi12010090

**Published:** 2021-01-16

**Authors:** Pilar Carreras, Itziar González, Miguel Gallardo, Alejandra Ortiz-Ruiz, Maria Luz Morales, Jessica Encinas, Joaquín Martínez-López

**Affiliations:** 1 CSIC, Spanish National Research Council, 28006 Madrid, Spain; iciar.gonzalez@csic.es; 2 Hospital 12 Octubre, Hematology Department, Research Institute i+12, 28040 Madrid, Spain; mgallardo@cnio.es (M.G.); mortizru86@gmail.com (A.O.-R.); mlmorales17@gmail.com (M.L.M.); jessi.encinas@gmail.com (J.E.); jmarti01@med.ucm.es (J.M.-L.); 3 CNIO, Spanish National Cancer Research Centre, Hematological Malignancies Research Unit, 28029 Madrid, Spain; 4 UCM, Medical Faculty, Complutense University Madrid, 28040 Madrid, Spain

**Keywords:** Microdroplets, Microfluidics, Stem cell culture, Hematopoietic stem cell

## Abstract

We previously reported a new approach for micromanipulation and encapsulation of human stem cells using a droplet-based microfluidic device. This approach demonstrated the possibility of encapsulating and culturing difficult-to-preserve primary human hematopoietic stem cells using an engineered double-layered bead composed by an inner layer of alginate and an outer layer of Puramatrix. We also demonstrated the maintenance and expansion of Multiple Myeloma cells in this construction. Here, the presented microfluidic technique is applied to construct a 3D biomimetic model to recapitulate the human hematopoietic stem cell niche using double-layered hydrogel beads cultured in 10% FBS culture medium. In this model, the long-term maintenance of the number of cells and expansion of hHSCS encapsulated in the proposed structures was observed. Additionally, a phenotypic characterization of the human hematopoietic stem cells generated in the presented biomimetic model was performed in order to assess their long-term stemness maintenance. Results indicate that the ex vivo cultured human CD34+ cells from bone marrow were viable, maintained, and expanded over a time span of eight weeks. This novel long-term stem cell culture methodology could represent a novel breakthrough to improve Hematopoietic Progenitor cell Transplant (HPT) as well as a novel tool for further study of the biochemical and biophysical factors influencing stem cell behavior. This technology opens a myriad of new applications as a universal stem cell niche model potentially able to expand other types of cells.

## 1. Introduction

Hematopoietic stem cells (HSCs) are located in the bone marrow and peripheral blood after mobilization and are the source of all blood cells through life [1,2,3]. Due to their ability to reconstitute the entire cellular compartment of the blood, HSCs are routinely transplanted to treat patients with life-threatening hematological disorders such as leukemia. However, often the clinical application of HSCs is limited by the fact that the number of patients in need exceeds the number of matching donors. Therefore, expansion of hHSCs ex vivo has been the focus of many efforts in research for a long time, as its successful in vitro expansion and maintenance would provide new opportunities for HSC therapies where there is a lack of this cells such as for HPT transplant. 

The natural environment where hematopoietic stem cells reside and maintain in vivo is the hematopoietic stem cell niche [4]. To date, there is no model that efficiently replicates the in vivo expansion of HSCs allowing cell proliferation while preserving their stem cell properties. When the hematopoietic stem cells are cultured ex vivo, they quickly initiate differentiation and lose their stem cell properties [5,6,7]. The maintenance and differentiation of the HSPC in its natural microenvironment is dependent on several factors, like the different supporting cell types, secreted factors, and the extracellular matrix [8,9,10]. For example, See et al. [10] developed a hHSC ex vivo culture system by supplementing HSC Expansion Medium with FLT3L, SCF, TPO, IL3, and IL6 (FKT36). Other attempts include the use of supporting cells as sources of the biochemical cues for the recapitulation of the hematopoietic stem cell niche. Bone marrow mesenchymal cells and endothelial cells have been repeatedly reported as suitable supporting cells favoring the maintenance and stemness of hHSCs [11,12,13]. Other factors reported to favor the stemness of hHSCs ex vivo include the direct contact between cells in the stem cell niche [14,15]. 

Several studies have reported the use of 3D scaffolds to recreate the hematopoietic stem cell niche as a biomimetic method to recapitulate the properties of the physiological niche and successfully culture long-term hHSCs [16,17]. Previous studies have reported the 3D hHSC culture up to 14 days using mesenchymal stem cells as supporting cells and scaffolds such as PEG hydrogels [18], collagen gels [19,20], fibrin polymers [21], or fibronectin conjugated polyethylene terephthalate (PET) matrices [22]. It has also been reported that a hypoxia gradient state in a co-culture system of hHSCs and hMSCs can favor the maintenance and quiescence of hHSCs [23,24,25]. Other studies reported a culture of up to 21 days by using a hydroxyapatite scaffold in a perfused system [26]. Most of these studies reported the stem cell culture in 3D aggregates or culture wells with no control over the shape or location of the feeder cells.

Another recent hHSC culture model was reported by Torisawa et al [27]. The group of Torisawa engineered an in vivo mouse bone marrow employing mouse hematopoietic stem cells. They achieved 7-day-long culture of hHSCs after transferring the mouse model to a microfluidic device. Another approach was described by Sieber et al. [28]. They integrated a platform of Multi-Organ-Chip (MOC) with a bone marrow model enabling the culture of the bone marrow model within a dynamic environment. They achieved the successful cultured for a time span of four weeks. Other successful studies on ex vivo long-term culture of HSCS were reported by Wilkinson et al. [29,30]. They described the development of an albumin-free culture system that supports the long-term ex vivo expansion of functional mouse HSCs. Using a simple platform, they demonstrated for the expansion of functional mouse HSCs ex vivo for more than one month.

The current limitations of the presented HSC-driven culture systems imply that HSCs generally die or differentiate rapidly in current ex vivo culture systems; therefore, there is still a need to recapitulate the natural hematopoietic stem cell microenvironment specially when using human primary stem cells. Whereas some of the most successful studies have used complex systems in which microfluidics was frequently part of the methodology, no previous studies have been reported using droplet microfluidics as a recapitulation of a human HSC niche model, and none of them for a time span of eight weeks.

Droplet-based cell culture models have drawn much attention due to their unique properties such that conventional culture systems cannot provide. The encapsulation of cells in specifically designed aqueous phase of a microfluidic system can provide profound understand of cell-to-cell and cell-to-extracellular matrix interactions that can also be used to study cell behaviors. A droplet-based cell culture system allows better control over confinement for culturing, maintaining, and analyzing cells, such as high-throughput screening [31].

Recently, we reported a method in which a double-layered hydrogel bead able to accommodate adult stem cells where controlled generation of the structure geometry, composition, and cell distribution was achieved [32]. We also recently described a successful technology to replicate and successfully culture ex vivo human Multiple Myeloma stem cells [33], therefore confirming the advantages of the droplet-based microfluidic platforms for the successful culture and maintenance of human primary difficult-to-culture stem cells. 

In this study, using the know-how already developed by our group, we report a droplet-based microfluidic platform able to generate double-layered hydrogel beads in a passive manner with tunable hydrogels for long term human hematopoietic stem cell culture. This model is comprised by two different hydrogel layers, the inner core containing human mesenchymal stem cells (hMSCs) in alginate, and the outer core containing bone marrow human hematopoietic stem cells (hHSCs) in Puramatrix, enabling successful hHSC culture for a time span of 8 weeks, without the need of added cytokines on the Dulbecco’s Modified Eagle’s Medium (DMEM) culture media. 

Hydrogel droplets (alginate) are produced by hydrodynamic focusing techniques and gelled by the circulation of the droplet between two laminar flows, one containing the cross-linking agent. This generated droplet is coated by another synchronized hydrogel bead (Puramatrix), generating on demand three dimensional niches for stem cell allocation by synchronizing droplet production rates. The combination of these processes of droplet generation and passive mixing without the use of external forces makes this technology suitable for the encapsulation of stem cells.

The aim of this study was the generation of a versatile, multicellular structure generation platform mimicking the human bone marrow and niche biology for HSPC sustainment and expansion, suitable to build an artificial HSC niche with biocompatible biomaterial scaffold, cell type without the use of any external force, and with a simple experimental set-up. Here, we present a novel device and methodology towards the replication of the human bone marrow niche model, allowing the long term encapsulation, maintenance of hHSC numbers in droplets and self-renewal of human hematopoietic stem cells. The presented culture system presents the advantages of providing on-demand control of the microenviromental conditions for the enclosed hHSCs, together with a simple microfluidic set-up. It is also a significant advantage the regular DMEM culture media used (without supplementary cytokines) and the minimum culture media changes required. 

## 2. Materials and Methods 

### 2.1. Materials/Chemicals 

Mineral oil (Sigma Aldrich, St. Louis, MO, USA) was used as the main oil carrier. Sudan red dye (Sigma Aldrich, St. Louis, MO, USA) was used to color the oil containing the cross-linking agent and visualize the interface between the two main carrier oil flows. Alginic acid sodium salt powder (Sigma Aldrich, St. Louis, MO, USA) was used to prepare the alginate solutions. The aqueous phase was composed of deionized water unless otherwise stated. Puramatrix, a commercially available self-assembling peptide gel (Becton Dickinson, Franklyn Lakes, NJ, USA) solution, was prepared at the corresponding concentrations by sonication of the gel for 30 min at 30 °C. Acetic acid (Sigma Aldrich, St. Louis, MO, USA) was used as the cross-linking agent. Nano-calcium carbonate powder was purchased from Skyspring Nanomaterials and used following the vendor protocol. 

### 2.2. hMSC Culture

The HS27a human bone marrow mesenchymal cell line (DSMZ) was cultured in DMEM with 10% FBS. Cell numbers at seeding were typically 1–3 million cells in a 75 cm^2^ flask. The media was changed every 48 h and the cells were kept at 37 °C and 5% (*v*/*v*) CO_2_. One to two weeks after seeding, cells were trypsinized, incubated for 30 min at 37 °C and 5% (*v*/*v*) CO_2_, centrifuged, and washed. 

### 2.3. Isolation of HSPCs

Bone marrow samples were collected from healthy donors after obtaining informed consent in accordance with the guidelines of the 12 Octubre Hospital ethics committee and the Declaration of Helsinki. Bone marrow mononuclear cells (PBMCs) were isolated by density gradient centrifugation (Ficoll-Paque™ PLUS, GE Healthcare, Little Chalfont, UK). PBMCs were washed with MACS buffer (pH 7.2) containing 0.5% bovine serum albumin (BSA) and 2 mmol/L ethylene diamine tetra acetic acid. The CD34+ cells were isolated using immunomagnetic columns (Miltenyi Biotec GmbH, Bergisch Gladbach, Germany) according to the manufacturer’s instructions. The procedure was performed twice to obtain a high purity of CD34+ cells.

### 2.4. hHSC Culture after Encapsulation

Beads were washed twice in PBS and cultured in Dulbecco’s Modified Eagle’s Medium (DMEM) (Biowest, Nuaillé, France) with 10% heat-inactivated fetal bovine serum (FBS) with no additional supplements. The simplicity of the media was selected so as to study just the influence of the presented culture technique over the maintenance and expansion of hHSCs.

### 2.5. Microfabrication

The microfluidic devices were designed using Solidworks CAD (version 24, Dassault Systems, Vélizy-Villacoublay, France) software. A computer numerical control (CNC) program was then created using Mastercam (CNC Software Inc., Tolland, CT, USA), according to the microfluidic circuit design and dimensions.

The outline of the device was extracted from a PMMA plastic sheet (MacMaster-Carr) using a Laser engraver (Universal Laser Systems, VLS 6.30, Scottsdale, AZ, USA). The plastic cut out was then taken to a CNC machine (HAAS Super Minimill), where the microfluidic circuit was created based on the Mastercam program. Milling conditions: milling direction, spindle speed, feed rate were optimized for surface quality. Finally, fluidic ports were created using a drill press (Ellis). 

**Figure 1:**
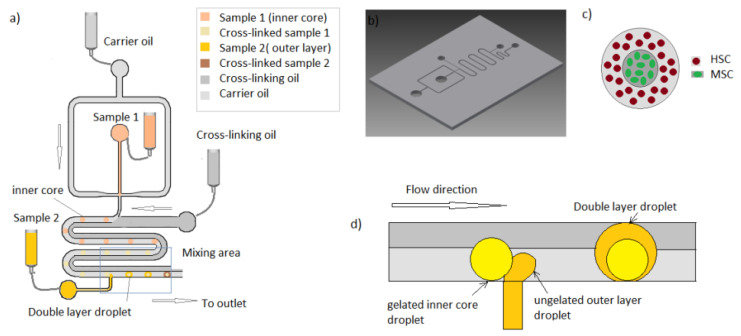
Schematic of the double-layer hydrogel generation device for hHSC long-term culture. (**a**) Microfluidic circuitry for the double-layered bead production (**b**) Computer-aided design (CAD) for the presented device. (**c**) Proposed model towards the recapitulation of the hematopoietic stem cell niche. (**d**) Detail of the process of layer deposition.

The PMMA device was sealed with a self-adhesive pouch (Opko diagnosis). Main channel dimensions are displayed in Figure 1 The device was connected to open barrels of 10 mL disposable plastic syringes (BD biosciences) via silicone tubing (ID 1.1 mm OD 2.16 mm) (Fisher scientific, Waltham, MA, USA). The driving force used for the experiments was gravity, so liquid pressure on the microdevice was controlled by the heights of the open syringes. Pieces of polytetrafluoroethylene (PTFE) tubing (OD 2 mm) (Cole Parmer, Vernon Hills, IL, USA) were used to interface the reservoirs to the chip.

### 2.6. Imaging

Fluorescence staining of the enclosed cells was performed by adding 3 μL of the correspondent antibody (CD34, CD11b, CD33, CD45, CD20, CD38, HLA-DR) (Becton Dickinson) per 2 mL of collected culture media and incubated at room temperature for 30 min. DAPI 0,1 M was added right before imaging (30 s to 1 min before imaging).

Fluorescence images were acquired using inverted laser scanning confocal microscopy (LSM510 META, Axiovert RT 200 M, Zeiss, Germany) equipped with an environmental chamber maintaining the cells under physiological condition, using the laser lines 405 nm and 488 nm. For all other experiments, a Motic Stereoscope (Motic, Richmond, BC, Canada) and Moticam 2.0 software (Motic) were used.

### 2.7. Microfluidic Platform

The microfluidic platform used was a double-layered hydrogel bead generator developed previously in your group [21]. The device operational is described in Figure 1. Experiments were run using alginate 1.6% wt/v containing calcium carbonate nanopowder 1.5% wt/v as a sample for the inner core. Microdroplets were generated by hydrodynamic focusing and flows driven by gravity means. The so-generated hydrogel droplets traveled through a meandering channel containing two laminar mineral oil flows. By the addition of acetic acid to the second laminar oil flow continuous phase, calcium ions were released after droplet formation by the diffusion of the acetic acid across the oil interface. Therefore, alginate microgel structures were generated in a repeatable and controlled manner. The so-generated droplets are then passed by a second inlet were a stream of second sample layer of Puramatrix 0.3% *v/v* droplets are generated in a synchronous way. Therefore, when the inner core droplet reaches the area of the second droplet production, the two droplets (one gelated and another ungelated) passively mix along the meander, generating a double layered hydrogen bead. 

Figure 1 shows the schematic of the microfluidic design and principle of operation.

## 3. Results

In order to perform long-term culture assays, preliminary experiments were designed to assess the shape and stability of generated beads after encapsulation. 

**Figure 2:**
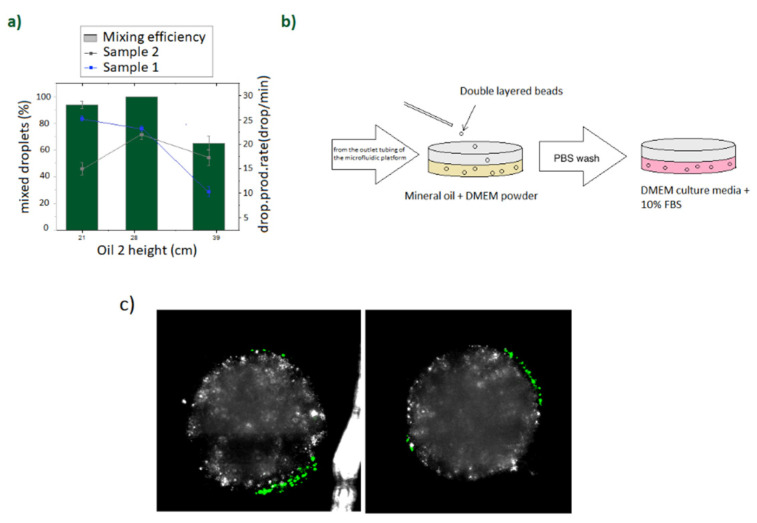
(**a**) Operational conditions for the long term double layer generation. (**b**) Schematic of the bead gelation and media transfer process. (**c**) Confocal imaging at the middle Z-plane of 2 different double Puramatrix- alginate beads at 4 h after encapsulation and media transfer using fluorescent latex beads. Green fluorescence corresponds to the fluorescent latex beads enclosed in the outer Puramatrix layer.

Premixed aqueous samples containing alginate (1.6 wt/v %) and calcium carbonate nanopowder (1.5 wt/v %) were prepared as scaffolds for sample 1, and Puramatrix (0.3% *v*/*v*) was prepared as scaffold for sample 2. The first experiments were run by adding fluorescent latex beads on the Puramatrix layer in order to assess its stability after gelation and during the collection process (Figure 2).

Concentrations for the gelation process were chosen to achieve a soft gelation state and favor cell–cell interaction. Experiments were run by fine tuning sample 1, sample 2, oil 1, and oil 2 flow rates by adjusting their corresponding barrel heights, so that sample 1 droplets will be regularly produced and cross-linked along the meander and passively mixed with a second generated droplet as described in previous work [22]. The specific conditions used for the presented experiments are displayed in Figure 2a.

Puramatrix is a commercial peptide hydrogel that gelates in presence of a salt or in contact with typical culture media. Therefore, in order to gelate the outer Puramatrix layer, beads were collected on a petri dish containing mineral oil premixed with DMEM powder. The schematic of the collection process is shown in Figure 2b. After collection, generated beads were maintained in the DMEM oil for 30 min, washed and transferred to a culture media flask for its further culture. 

The generated double-layered beads were comprised by an inner core of gelated empty alginate and a fine coating Puramatrix outer layer enclosing the fluorescent latex beads. The operational conditions for the device were chosen by varying the height of the cross-linking oil to establish the optimal flow rate to achieve a continuous mixing of droplets (Figure 2a). Collected structures were cultured during few hours as a preliminary trial to investigate bead shape and stability (Figure 2). After the process of gelation and transfer to DMEM culture media, beads were maintained during 4 h at the culture media and imaged under confocal microscopy. It was confirmed that the encapsulated latex beads were located and maintained at the outer Puramatrix layer without transferring to the inner layer of the bead, therefore demonstrating the suitability of the double-layered structure as a compartmentalized model of the hematopoietic stem cell niche. Once the stability of the proposed structure after collection was assessed, the suitability of the presented method for hHSCs cell encapsulation was demonstrated by accommodating primary patient-derived human hematopoietic stem cells in the outer layer of the double-layered bead and supporting hMSCs in the inner core.

For this purpose, and prior to resuspension in PBS and mixture in premixed alginate emulsification, human mesenchymal stem cells were routinely cultured and trypsinized. A sample containing 24 million hMSC cells per milliliter was prepared. Forty-eight-million hMSCs were resuspended in 200 microliters of PBS and mixed in 1.8 mL of the sample 1 scaffold prior to sample injection. Following this same procedure, 5 million hHSC cells were resuspended in 200 microliters of PBS and mixed in 800 microliters of the scaffold for sample 2 before sample injection.

The generated cell-laden structures were collected and washed to remove residual acid. FITC-conjugate anti-human CD34+ which fluoresces green identifying human Hematopoietic stem cells and DAPI 0,1 M which binds to the DNA of dead membrane compromised cells were employed to visualize the distribution of hHSCs alive and dead cells right after encapsulation.

**Figure 3:**
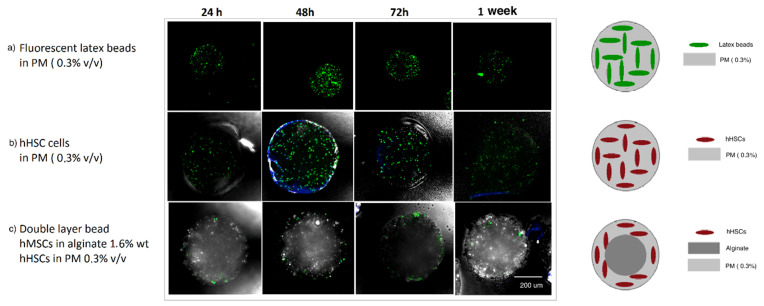
Confocal images at 24, 48, and 72 h and one week culture of (**a**) latex beads in Puramatrix (0.3% *v*/*v*) (in green), (**b**) primary FITC-CD34+/DAPI labeled hHSCs in PM (0.3% *v*/*v*), and (**c**) double-layered beads containing FITC-CD34+/DAPI labeled hHSC in Puramatrix (0.3% *v*/*v*) in the outer layer and hMSCs in alginate (1.6% wt/v) in the inner layer (unlabeled). Black and white unlabeled fractions correspond to calcium carbonate or alginate particles A double-layered bead without hHSC in the outer layer is displayed in Figure S1.

During this experiment, single Puramatrix droplets containing fluorescent latex beads were also generated and cultured for a week to asses Puramatrix cross-linking procedure and stability over a week (Figure 3). Single Puramatrix droplets containing human HSCs were also generated and cultured as a control and to assess the cell-containing Puramatrix cross-linking procedure and stability. All beads were collected and cultured in DMEM with 10% heat-inactivated fetal bovine serum (FBS) over 7 days. The medium was refreshed each week. Cultures were incubated at 37 °C in a humidified atmosphere with 5% CO_2_ for 8 weeks. 

The FITC-CD34+ (green)-DAPI (blue) assay was performed to asses viability and stemness of the enclosed hHSC cells. Three microliters of FITC-CD34+ antibody was added per 2 mL of culture media and incubated for 30 min at room temperature. 

Results are shown in Figure 3. A maintained population of CD34+ cells in both single- and double-layered structures was observed after a week, indicating the appropriateness of both Puramatrix and the combined double layer scaffold to allocate human hematopoietic stem cells for a one week cell culture assay.

**Figure 4:**
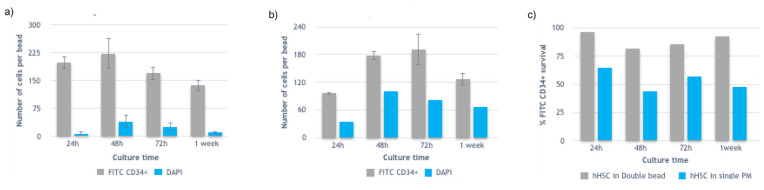
(**a**) Average number of CD34+cells per bead and dead cells for different time points up to a week in a double-layered hydrogel structure. (**b**) Average number of CD34+ cells enclosed per bead and dead cells for different time points up to a week in a single Puramatrix bead. (**c**) Comparison of CD34+ survival rate for double-layered beads and single beads.

Experiments in Figure 3b,c were repeated and imaged over a week. Four to five beads were imaged in different Z-planes and analyzed (double-blind counted) for each condition using ImageJ software. Z-planes were chosen to include the maximum number of cells and overlapped to avoid double cell counting. Two different confocal microscopy channels were used to account for the CD34+ (green) marked and DAPI (blue) marked human hematopoietic stem cells. Results are shown in Figure 4.

Beads from the same cell sample and same experiment were collected both as a single Puramatrix droplet production and after mixing with the inner core and forming a double-layered bead. Therefore, we considered that a comparable amount of hHSCs were enclosed in the generated beads of the same experiment, and therefore that a single bead of Puramatrix contained a comparable number of hHSC cells as those in a double layered bead of the same batch. Therefore, results indicate that in the first 24 h there was a fold increase in total cell numbers in the double layered beads compared to the single Puramatrix beads. At 48 h, hHSCs cells seemed to expand in both control cases, whereas cells enclosed in the Puramatrix control beads showed a higher cell mortality rate. It was also noticed a decrease of the expansion rate of hHSCS encapsulated in double layered beads at 72 h while hHSC cell numbers enclosed in Puramatrix increased. Additionally, at that same time point, the cell mortality rate was higher for cells in Puramatrix than for those encapsulated in double layered beads. For both controls, the expansion rate trend decreased between 72 h and a week culture time.

From this study it was concluded that although both types of beads succeeded in maintaining and expanding hematopoietic stem cells over a week in a DMEM 10% FBS culture media without any additional supplement, the Puramatrix and alginate matrix represented the optimal structure for recapitulating the hHSC stem cell niche. Therefore, the most suitable double layered bead constructed via Microniche technology was determined as to be composed by the inner layer alginate 1.6% wt/v and outer layer Puramatrix 0.3% *v*/*v*.

Once it was demonstrated the possibility of maintaining and expanding hHSCs in the proposed structure, the following experiments were directed towards improving the survival, expansion rate and number of collected beads (more than 100 beads per condition) to assess the maximum time that hHSCs could be cultured with the presented methodology. 

For this purpose, and prior to resuspension in PBS and mixture in premixed alginate emulsification, human mesenchymal stem cells were routinely cultured and trypsinized. Twenty-four-million hMSC cells were resuspended in 200 microliters of PBS and mixed in 1.8 mL of the sample 1 scaffold prior to sample injection. Following this same procedure, 2 million hHSC cells were resuspended in 200 microliters of PBS and mixed in 800 microliters of the scaffold for sample 2 before sample injection.

The experimental sample was collected from a different patient and in different conditions compared to the previous experiment. The procedure for the generation, collection, and culture of the structures was performed as described before. In order to enhance cell survival, collected beads were washed three times with PBS before being transferred to the culture media in order to minimize the amount of residual acidic oil before their culture in DMEM media. Time between bead collection and transfer to culture media was also minimized. Media changes on 2 mL wells containing 6–7 beads were performed every week and collected DMEM from the corresponding culture was frozen at −80 C for its further analysis. Following these conditions, beads were imaged and maintained up to eight and a half weeks.

Four to five beads were imaged in different Z-planes and analyzed (double-blind counted) for each condition using ImageJ software. Z-planes were chosen to include the maximum and total number of cells and overlapped to avoid double counting. Beads were also chosen so that clusters of cells were not present on the bead. When clusters of cells were included, an approximation of the number of cells contained in the cluster was approached based on the maximum size of a positive cell located on the same Z-plane. Two different confocal microscopy channels were used to account for the CD34+ (green) and DAPI (blue) marked human hematopoietic stem cells (Figure 4).

**Figure 5:**
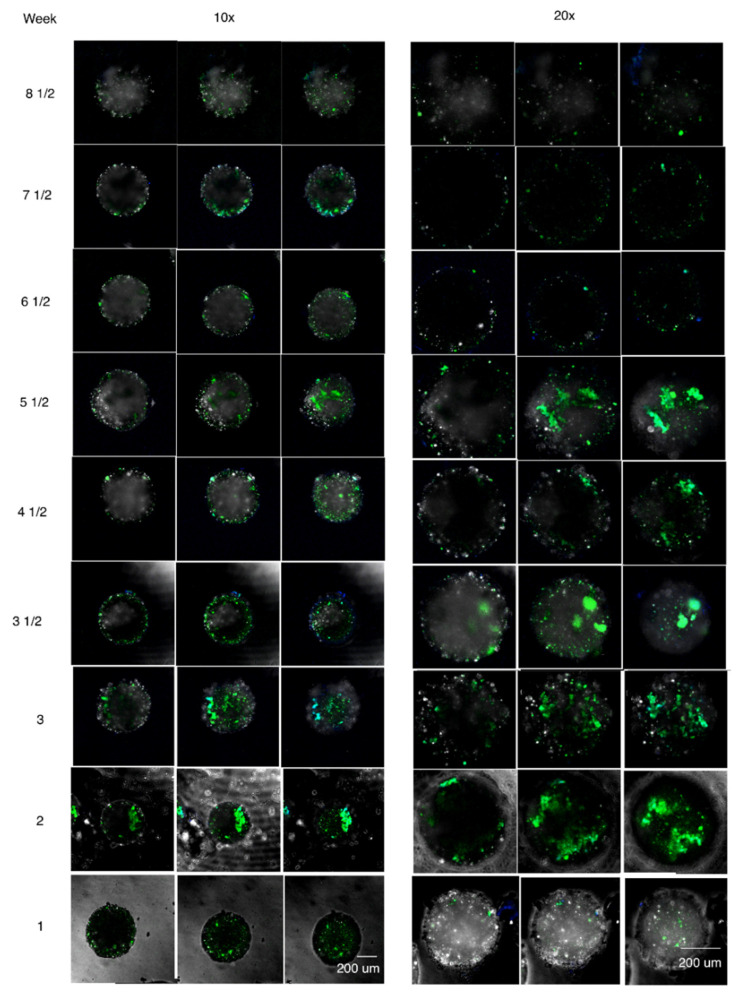
Confocal images examples of cell viability FITC-CD34+ (green) and DAPI (blue) experiments using double-layered bead structures containing hMSCs in the inner core and hHSCs in the outer layer up to 8 and a half weeks in culture. Images represent three middle Z-planes of the bead. Left: 10× objective and Right: 20× objective. Figure S2 contains additional images representative of the process. Figure S3 contains a representative total fluorescence analysis for this images.

Results of the collected double-layered beads for the different time points and Z-planes are shown in Figure 5. Figure 5 shows the confocal images of three different Z-planes focused along selected beads using both 10× and 20× amplification.

During the time spam of the presented long-term culture experiment, generated beads were also tested for different lineage hematopoietic markers to assess its hematopoietic differentiation.

Phenotypic characterization experiments were designed so that cells were marked in two different colors using FITC CD34+ green and a complementary marker in blue. No DAPI marker was added to this experiment due to confocal microscopy limitations. FITC (green)-marked cells, double-marked cells (green and blue), and phenotype-marked (blue) cells were counted to assess the state of the enclosed cells. 

The chosen markers for this series of experiments were CD11b and CD33 as representatives of myeloid differentiation; CD20, CD38, and HLA-DR for lymphoid differentiation; and CD45 as a pan-leukocyte marker. These experiments were designed in order to enlighten information about the maturation, differentiation, or variation of the cultured stem cell phenotype (CD34+). Three milliliters of the correspondent antibody was added per 2 mL of culture media and incubated for 30 min at room temperature. Beads were extracted from the antibody containing culture media, washed twice with PBS, and transferred to a 10% FBS DMEM culture media before confocal imaging. 

**Figure 6:**
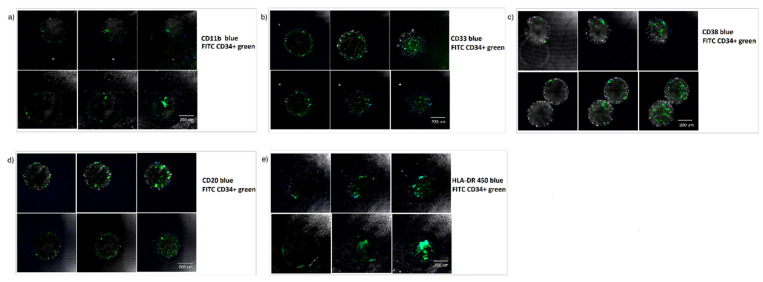
Impact of the Microniche culture system on HSPC phenotypic characterization at week 4. Double-layer bead (inner core hMSCs in alginate and outer core hHSCs in PM) tested for several hematopoietic markers: CD11b macrophages, CD20 B lymphocytes, CD33 myeloid, CD38 B lymphocytes, and HLA-DR T cell. (**a**) CD11b (blue)-FITC CD34+ (green). (**b**) CD20-FITC CD34+ (green). (**c**) CD33-FITC CD34+ (green). (**d**) HLA DR-FITC CD34+ (green) at week 3 and ½. (**e**) CD38 (blue) FITC CD34+(green).

At week 4, a set of the long-term cultured beads was separated, incubated with the corresponding phenotype markers and visualized under confocal microscopy (Figure 6). It could be observed that most cells remained undifferentiated, while some beads show a slight lymphoid differentiation (CD20, CD38) and a slight myeloid differentiation (CD33). It was also detected an intermediate stem cell population generation (CD34+/HLA-DR+).

**Figure 7:**
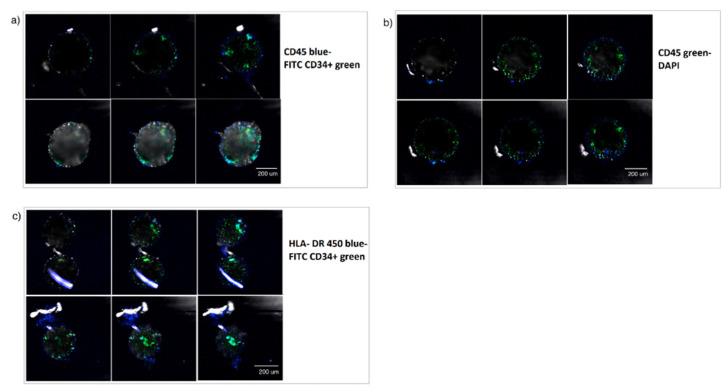
Impact of the Microniche culture system on hHSCs phenotypic characterization. Double-layer bead (inner core hMSCs in alginate and outer core hHSCs in PM) tested for (**a**) CD45 (blue)-FITC CD34+ (green), (**b**) CD45 (green)-DAPI (blue) at week 6 and ½, and (**c**) HLA-DR (blue)-FITC CD34+ ( green).

Additionally, at week 6, a second set of the cultured beads were separated and incubated with the hematopoietic marker CD45 in both green and blue and compared to DAPI, and HLA-DR 450 in blue to obtain additional information on the phenotypic characterization of the enclosed cells. Figure 7 shows some examples of the images obtained under confocal microscopy. 

It could be observed that the encapsulated hHSCs started to differentiate significantly without reaching maturation, forming intermediate phenotypes CD34+/CD45+ y CD34+/HLA-DR+ as also observed at week 4. The phenotypic characterization experiments also demonstrated that the cells were still functional and capable of differentiating into their various progenies at the selected time points, indicating a stable maintenance of the cell numbers of functional hHSPCs within the presented model. 

**Figure 8:**
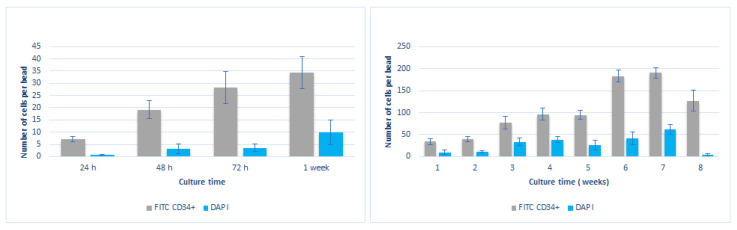
Left: Number of cells per bead for double-layered beads containing hMSC in 1.6% wt/v alginate in the inner layer and hHSCs in 0.3% *v*/*v* PM in the outer layer in the first week of culture using FITC CD34+ and DAPI. Right: Number of cells per bead for double layered beads containing hMSCs in 1.6%wt/v alginate and hHSCs in 0.3% PM for 8 weeks of cell culture.

Images were analyzed as described before and enclosed cultured cells were counted over a minimum of ten beads per condition, taking into account cells imaged at least in four planes along the Z-plane in order to quantify the number of positive FITC- CD34+ cells and DAPI during the eight week culture (Figure 8). 

Figure 8 shows that CD34+ cell numbers augmented progressively during the first 24, 48, and 72 h. The number of DAPI positive cells also augmented progressively while staying much lower than the CD34+ counted cells. A significant increase of CD34+ cells was detected at weeks 6 and 7, reaching its maximum at week seven and starting to decrease till the end of the reported period (8 weeks). These results indicated the great potential of the presented methodology to recapitulate a suitable microenvironment to maintain and expand hHSCs. Although Figure 8 shows that we see a clear expansion of the enclosed hHSC cells for 8 weeks, this was observed in a small set of experiments (*n* = 4–5). Therefore, to be able to make a strong conclusive remark, a larger sample size is required to achieve a strong statistical power analysis. 

**Figure 9:**
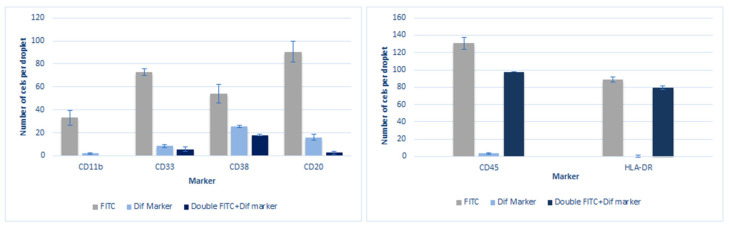
Number of cells per bead for double-layered beads containing hMSC in 1.6% wt/v alginate in the inner layer and hHSCs in 0.3% *v*/*v* PM in the outer layer for (Left) FITC-CD34+ and phenotype markers Cd11b, CD33, CD38, and CD20 at week 4. (Right) FITC-CD34+ and phenotype markers CD45 and HLA-DR at week 6.

Cultured cells were also counted for the experiments measuring the impact of the microniche structure on the phenotypic characterization of the enclosed hHSC cells (Figure 9). Results are shown below:

Although the cell counting method was just an approximate method to estimate the cell expansion inside the proposed beads, the results clearly indicate that there was a fold increase of CD34+ cell numbers during the reported period.

Additional studies were performed to detect and identify hematopoietic stem cell cytokines in the collected culture media. This analysis aimed to retrieve some information which could enlighten the mechanisms underlying the process of cell maintenance and expansion in the generated beads for further studies. This analysis was performed using culture media from double hHSC and hMSC beads experiments collected as a sample. Samples collected at different time points were analyzed using a hematopoietic cytokine kit (Legenplex, human Hematopoietic stem cell panel, Biolegend). 

To perform this assay, six serial dilutions were prepared from a stock solution to obtain the standard curve. Each well of the panel was filled with buffer and 25 microliters of the stock solution (to obtain the standard curve) or 25 microliters of the culture media to study. Twenty-five microliters of mixed beads was also added to all panel wells and incubated 2 h at 800 rpm. Then, the panel was centrifuged at 1050 rpm for 5 min and washed with 200 micrometers of wash buffer. This last step was repeated twice. The detection antibodies (25 microliters per well) were added to the wells and the panel was incubated again for 1 h at 800 rpm. Twenty microliters of streptavidin-PE was added to the wells and incubated again for 30 min at 800 rpm. The supernatant was removed and samples were washed twice with 200 microliters wash buffer. Samples were then resuspended in 150 microliters of wash buffer and analyzed by citometry using Legendplex software.

As a result of this analysis, it was found a significant increase on the concentrations of IL-34 cytokine. At some time points it was also observed a slight increase IL-11 and IL-7 (complete cytokine data shown in Figure S4), corresponding with the observed phenotypic characterization results. Other cytokines were not detected significantly at the collected media probably due to the low cell concentration per bead and per milliliter of culture media. 

**Figure 10:**
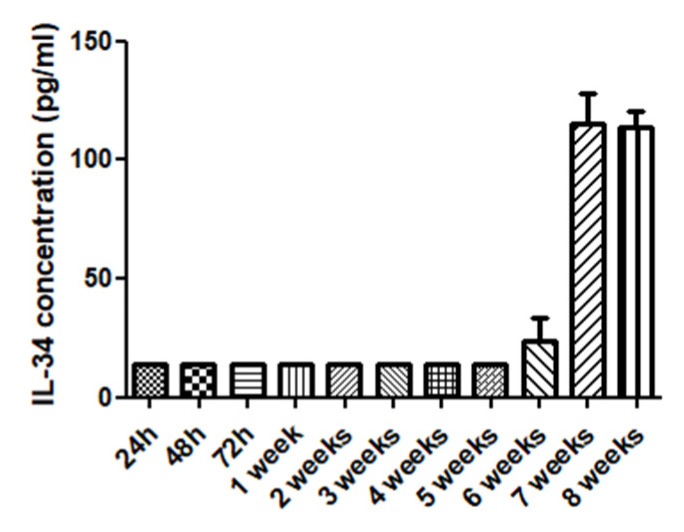
Cytokine IL-34 concentration analysis in double-layered hHSC-hMSC beads culture media and culture time up to 8 weeks.

A Kruskal–Wallis test was performed over the data of cytokine IL-34 with significant results (*p* = 0.0226). IL-34 cytokine analysis is shown in Figure 10. This result could trigger further research to investigate if the significant increase of IL-34 on the culture media could be an indicator of the maintenance and stemness of hHSCs.

## 4. Discussion

Hematopoietic stem cells have great potential for regenerative medicine applications, and its maintenance and expansion have been the object of many studies where they are applied to improve the current HPT treatments. Recent efforts have been made towards developing in vitro systems including three-dimensional culture systems allowing the maintenance and expansion of primary human hematopoietic stem cells, but this still remains a challenge. 

In the current manuscript, we describe a novel protocol to successfully maintain and expand human primary HSCS from bone marrow in long-term cultures. The presented procedure represents a novel droplet-based culture system for the encapsulation, maintenance and expansion of human primary hematopoietic stem cells. This novel technology is based on a passive mixing principle and a gelation system using a double laminar oil flow where only one contains the cross-linking agent allows the mild generating of double-layered hydrogel beads containing stem cells on demand. The soft consecutive coating of the inner core with a second layer without exposing the encapsulated cells to external forces that might reduce their viability constitutes the main technology advance towards a platform for 3D stem cell encapsulation in general and in particular for the recapitulation of the Hematopoietic stem cell niche. 

The obtained results indicate that hHSCs viability is successfully maintained over at least eight weeks inside the generated microniches by microfluidic techniques. The so-cultured cells showed an increasing significant expansion of CD34+ cells up to week seven, after which CD34+ cell numbers started to decrease. 

Patient-derived cultured cells in droplets were also phenotypically characterized to assess its viability and differentiation capacity. It was observed that the great majority of the enclosed cells remained stem, with very small subpopulations of differentiated cells. The cytokine study also showed a significant increase of IL-34 cytokine on the collected culture media. These overall results suggest that most of the ex vivo maintained human Hematopoietic stem cells enclosed using the presented technology remain in its original stem state and can be cultured and expanded in the long term, therefore opening opportunities to both improve HPT treatments or be used as a therapeutic platform.

Additionally, the presented device and procedure is simple and low cost and does not require the supplement of any supporting cytokines on the culture media. In this study, generated beads are cultured in typical DMEM culture media supplemented with 10% FBS.

From the 3D culture device perspective, our results demonstrate that the presented technology can recapitulate the extra cellular environments to help reproduce the in vivo conditions of cells that are difficult to culture, opening new lines of research.

In particular, Microniche technology has successfully achieved long-term human HSCs survival and expansion by enclosing these cells in a double-layered hydrogel structure with supporting human Mesenchymal stem cells (hMSCs) for a time span of 8 weeks. The possibility of maintaining ex vivo hematopoietic stem cells from bone marrow for more than eight weeks represents a significant advance towards providing Hematopoietic Progenitor cell Transplant (HPT) improvements as well as a novel tool for further study of the biochemical and biophysical factors influencing stem cell behavior. This technology opens a myriad of new applications as a universal stem cell niche model potentially able to expand other types of cells.

## Data Availability

Not applicable.

## Supplementary Materials

The following are available online at https://www.mdpi.com/2072-666X/12/1/90/s1, Figure S1: Confocal Microscopy a 3-channel (green, bright field and blue channel) picture of an extracted bead con-taining an inner core of alginate (1.6%wt) containing 24 millions per ml hMSCs cells and an empty outer layer (without cells) incubated for 30 min with FITC-CD34+ and DAPI; Figure S2: Confocal Microscopy Z-plane pictures of extracted beads containing an inner core of alginate (1.6%wt) containing hMSCs cells and an outer layer of hHSCs incubated for 30 min with FITC-CD34+ and DAPI for 1,2,4,6 and 8 weeks time culture; Figure S3: Total fluorescence analysis of the green channel corresponding to weeks 1.2.4.6 and 8 and for extracted beads contain-ing an inner core of alginate (1.6%wt) containing hMSCs cells and an outer layer of hHSCs incubated for 30 min with FITC-CD34+ and DAPI for 1,2,4,6 and 8 weeks time culture; Figure S4: Cytokine kit (Legenplex, human Hematopoietic stem cell panel, Biolegend) concentration analysis in double layered hHSC-hMSC beads culture media and culture time up to 8 weeks.
