# Supplementary material for: Long-Term Human Hematopoietic Stem Cell Culture in Microdroplets
Source: Micromachines (Basel). 2021 Jan 16;12(1):90. doi: 10.3390/mi12010090 (PMC7830102; doi:10.3390/mi12010090)
Supplement: Supplementary file 1 [file micromachines-12-00090-s001.pdf]

micromachines

Supplementary Materials: Long-Term Human Hematopoietic Stem Cell Culture in Microdroplets

Pilar Carreras, Itziar Gonzalez, Miguel Gallardo, Alejandra Ortiz-Ruiz, Maria Luz Morales, Jessica Encinas and Joaquín Martínez-López

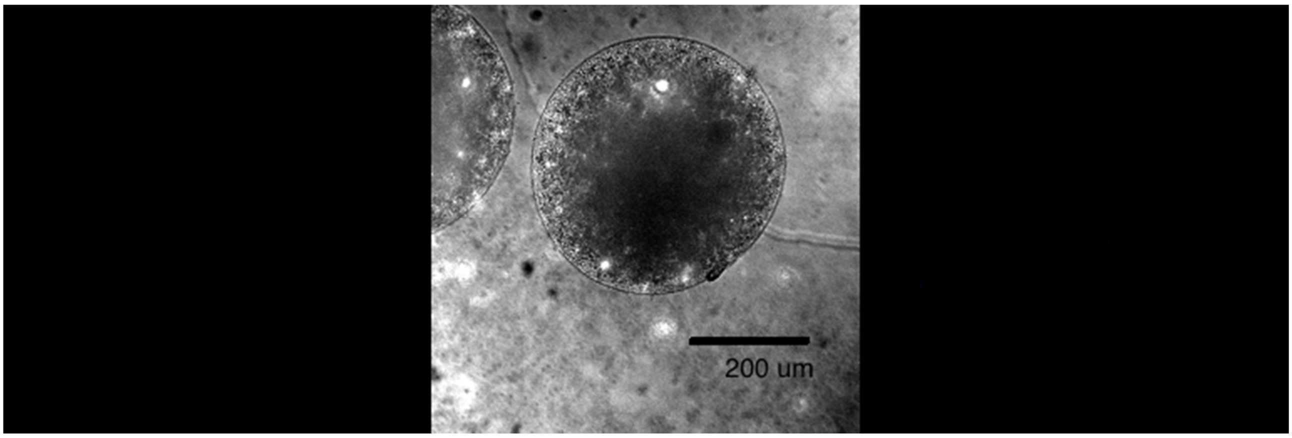

Figure S1. Confocal Microscopy a 3-channel (green, bright field and blue channel) picture of an extracted bead containing an inner core of alginate (1.6% wt) containing 24 millions per ml hMSCs cells and an empty outer layer (without cells) incubated for 30 min with FITC-CD34+ and DAPI.

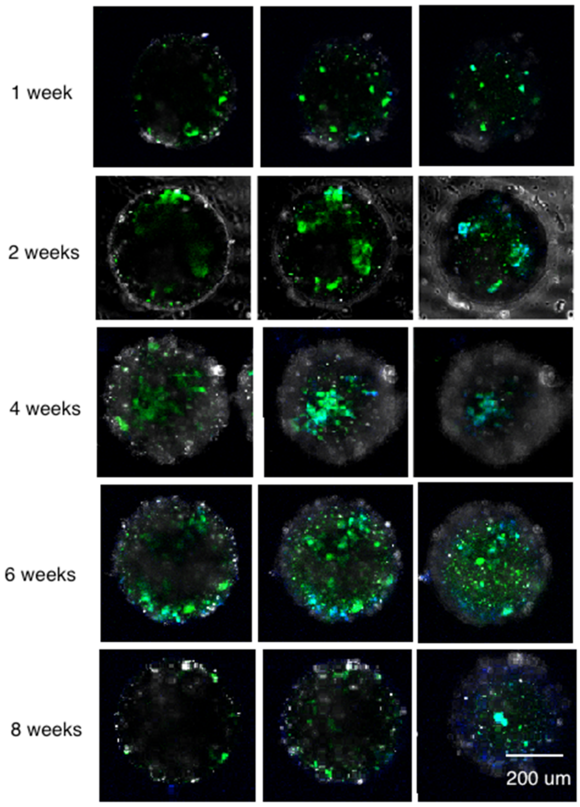

Figure S2. Confocal Microscopy Z-plane pictures of extracted beads containing an inner core of alginate (1.6% wt) containing hMSCs cells and an outer layer of hHSCs incubated for 30 min with FITC-CD34+ and DAPI for 1,2,4,6 and 8 weeks time culture.

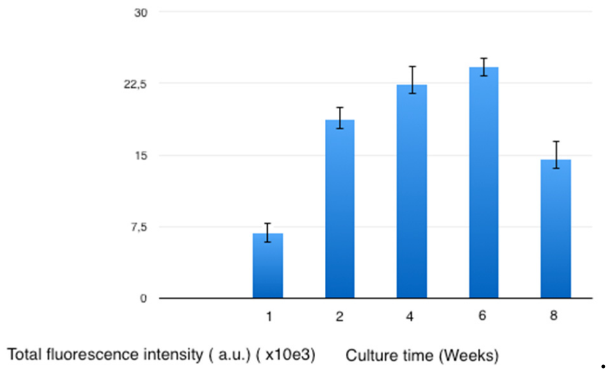

Figure S3. Total fluorescence analysis of the green channel corresponding to weeks 1,2,4,6 and 8 and for extracted beads containing an inner core of alginate (1.6% wt) containing hMSCs cells and an outer layer of hHSCs incubated for 30 min with FITC-CD34+ and DAPI for 1,2,4,6 and 8 weeks time culture.

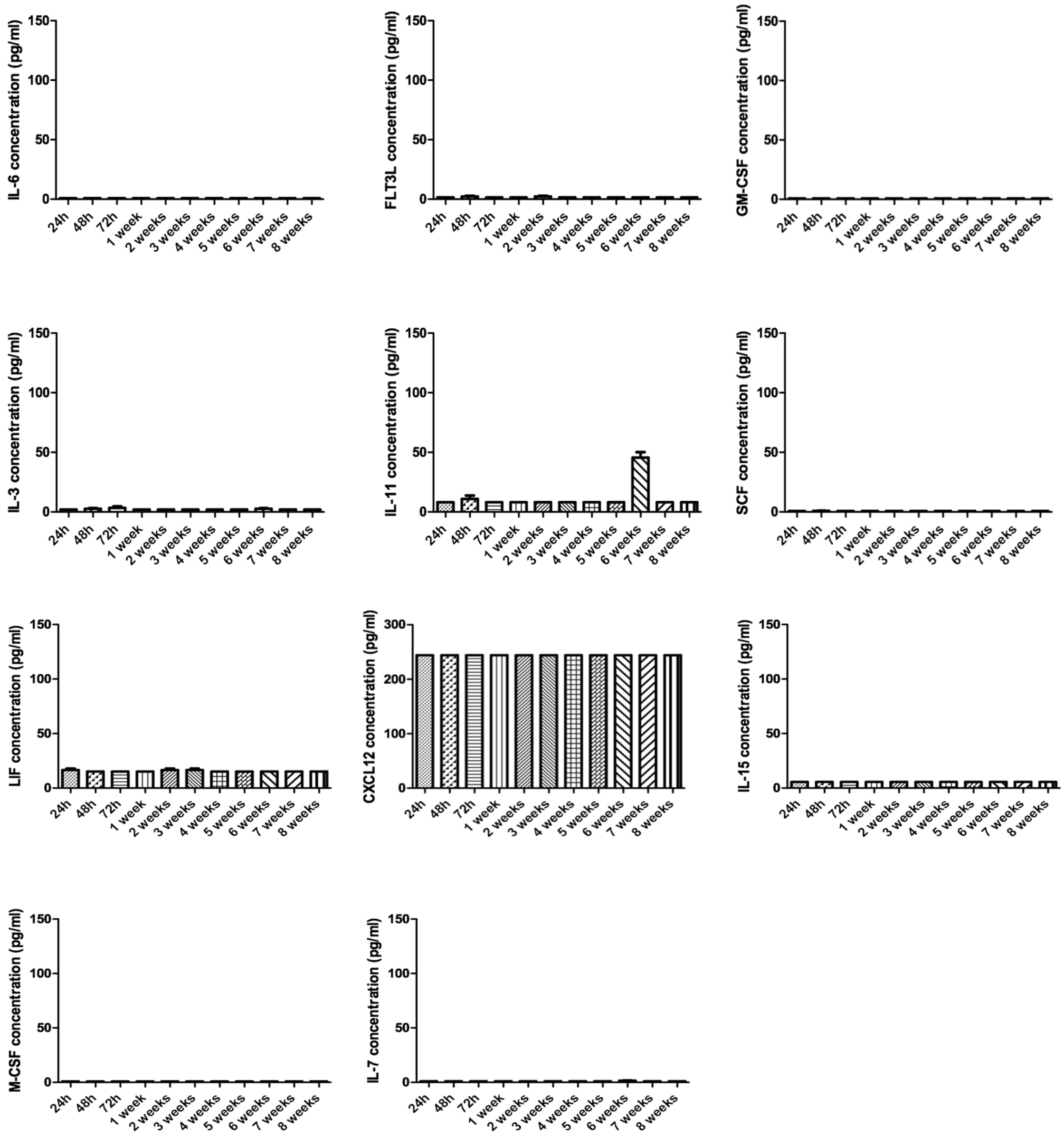

Figure S4. Cytokine kit (Legenplex, human Hematopoietic stem cell panel, Biolegend) concentration analysis in double layered hHSC-hMSC beads culture media and culture time up to 8 weeks.
